# The enigmatic role of Mfd in replication-transcription conflicts in bacteria

**DOI:** 10.1016/j.dnarep.2019.102659

**Published:** 2019-09

**Authors:** Mark Ragheb, Houra Merrikh

**Affiliations:** a Molecular and Cellular Biology Graduate Program and Medical Scientist Training Program, University of Washington, Seattle, WA, USA; b Department of Biochemistry, Vanderbilt University, Nashville, TN, 37205, USA; c Department of Pathology, Microbiology, and Immunology, Vanderbilt University Medical Center, Nashville, TN, 37232, USA

**Keywords:** RNAP, RNA polymerase, DSB, double-stranded DNA break, NER, nucleotide excision repair, TCR, transcription-coupled repair, AMR, antimicrobial resistance, Replication-transcription conflicts, Replisome-RNAP conflict resolution factors, Mfd

## Abstract

Conflicts between replication and transcription can have life-threatening consequences. RNA polymerase (RNAP) is the major impediment to replication progression, and its efficient removal from DNA should mitigate the consequences of collisions with replication. Cells have various proteins that can resolve conflicts by removing stalled (or actively translocating) RNAP from DNA. It would therefore seem logical that RNAP-associated factors, such as the bacterial DNA translocase Mfd, would minimize the effects of conflicts. Despite seemingly conclusive statements in most textbooks, the role of Mfd in conflicts remains an enigma. In this review, we will discuss the different physical states of RNAP during transcription, and how each distinct state can influence conflict severity and potentially trigger the involvement of Mfd. We propose models to explain the contradictory conclusions from published studies on the potential role of Mfd in resolving conflicts.

## 1 Introduction

DNA replication and transcription must occur in a timely and accurate fashion in all organisms. Individually, each one of these tasks is a remarkable undertaking. It is even more striking to consider that replication and transcription utilize the same DNA template, yet, the machineries responsible for these respective processes must not impede each other’s function. While cells are remarkably efficient at coordinating replication and transcription, there are unavoidable encounters between the replication machinery (the replisome) and RNAP polymerase (RNAP). The encounters between these machineries, termed replication-transcription *conflicts*, come in two different flavors: head-on (if a gene is encoded on the lagging stand of replication) or co-directional (if a gene is encoded on the leading strand of replication). Conflicts can lead to various detrimental outcomes, including replication fork stalling, double-stranded DNA breaks (DSBs), and mutations. In general, these events are more severe in the head-on orientation. For comprehensive insight into the consequences of such conflicts in both prokaryotes and eukaryotes, see the following reviews: [[1], [2], [3], [4], [5], [6], [7]].

Cells have a complement of factors to deal with replication-transcription conflicts and their consequences. Research has largely focused on proteins that help replication progression at these encounters. These include proteins implicated in replication restart [8], replication fork reversal [9], DNA break repair [10], as well as enzymes that help deal with R-loops (three stranded structures composed of an RNA:DNA hybrid and a complementary, single DNA strand) [[11], [12], [13]].

As with most conflicts, there are two sides to the story; in the conflict between replication and transcription, it is the role of the latter that is less well understood. Various RNAP associated factors help remove RNAP from the sites of protein roadblocks [[14], [15], [16]] or bulky DNA lesions [[17], [18], [19]]. Although there have been suggestions that these factors may also mitigate conflicts, in most cases concrete evidence for conflict resolution by these proteins is lacking. We will discuss in detail how different states of RNAP can impact replisome progression, and largely focus on one highly conserved RNAP associated bacterial translocase, Mfd. We will summarize the results of the main studies on the role of Mfd and provide explanations for why these studies may have come to different conclusions.

## 2 The various states of RNAP and the predicted impact on DNA replication

RNAP can encounter the replisome in various, distinct states. During unimpeded transcription, RNAP adopts a long-lived active complex,which can readily incorporate incoming nucleotides [[20], [21], [22]]. Impediments to transcription, such as protein roadblocks and nucleotide starvation, can induce distinct conformational changes to RNAP, rendering the enzyme inactive. Functionally, inactive RNAPs are unable to proceed with transcription elongation, and can at times backtrack on DNA [[23], [24], [25], [26], [27]]. In the backtracked state, RNAP slides reversibly backwards on DNA, becoming an inactive, highly stable complex. Extensive RNAP backtracking leads to the full arrest of the complex [28]. Evidence from Dutta et al. [29] suggests that backtracked RNAPs promote DSBs at replication-transcription conflict sites. Therefore, conditions that promote RNAP backtracking are also likely to promote more deleterious conflicts.

## 3 The impact of transcription dynamics on conflicts

Most of our understanding about conflicts has focused upon transcription elongation. The process of transcription is dynamic, and includes three stages: initiation, elongation, and termination. Conflict severity in general, and gene orientation-specific effects, may largely depend upon the stage of transcription.

*Transcription initiation*: At the initiation stage, the RNAP holoenzyme (the RNAP core enzyme consisting of five subunits (β′, β, α^I^, α^II^, and ω) and a sigma factor subunit in bacteria [30]) recognizes the upstream promoter binding sites, melts the DNA to form a transcription bubble, and begins synthesizing RNA [[31], [32], [33]]. Subsequently, RNAP goes through multiple rounds of abortive initiation, generating a series of short RNA products until RNAP finally escapes the promoter site to form the elongation complex [34,35]. The rate of RNAP translocation during initiation is significantly slower than that during elongation and data shows that RNAP spends at least ˜50-fold greater time at an initiation site relative to any other position within the transcription unit [33,36]. Initiating RNAPs can also form stable, backtracked complexes [33]. However, it is not yet clear whether conflicts between initiating RNAPs and the replisome occur, and if so, whether they are fundamentally different than those with elongating RNAPs.

While it is plausible that the replisome encounters initiating RNAPs at some frequency during conflicts, studies using the bacterial transcription inhibitor rifampicin suggest that transcription elongation can directly impede replication. Rifampicin arrests RNAP at the transcription initiation phase [37,38], rapidly clearing genes of elongating RNAPs. There is evidence that clearing transcription elongation complexes using rifampicin completely rescues stalled replisome complexes, and promotes replication progression [8]. Additionally, mutants of RNAP that destabilize transcription elongation complexes also reduce conflict severity [8,11]. These studies have revealed that transcription elongation plays a critical role in conflicts. Therefore, elongating and not promoter-associated RNAPs are likely the major obstacle to replisome progression.

*Transcription elongation*: During elongation, RNAP forms a stable, processive complex on DNA, moving at a speed of approximately 10–100 nucleotides/second [[39], [40], [41]]. Since roadblocks to transcription reduce the rate of elongation, conflicts between the replisome and elongating RNAP are not singularly defined.

The rate of elongation can be altered by various factors. Naturally occurring RNAP pausing decreases the rate of elongation and is perhaps the rate-limiting step in transcription [42]. Moreover, the presence of roadblocks such as DNA lesions or DNA-bound proteins (e.g. an upstream stalled RNAP or a repressor protein) can also impede elongation [43] and induce backtracking [44,45]. Inducing RNAP arrest is thought to exacerbate conflicts. Indeed, work from Trautinger et al. [18] shows RNAPs stalled by DNA damage increase the severity of conflicts with replication, and require factors that remove RNAP, such as Mfd, for cell viability.

Translation can also enhance transcription elongation, as the coupling of these two processes in bacteria helps RNAP processivity by reducing backtracking [29]. This suggests that, although indirect, translation can also impact conflict severity through its influence on RNAP elongation. Only one study has investigated the impact of ribosomes on replisome progression. Inhibiting translation at a conflict site (and subsequently enhancing backtracking), was shown to increase the severity of the conflict [29].

Arrested transcription elongation is only one of multiple factors during transcription that can significantly alter the severity of replication-transcription conflicts. In particular, as previously discussed, gene orientation is a major determinant of conflict severity given that head-on replication-transcription conflicts are far more deleterious than co-direction conflicts. It is also thought that higher levels of transcription and longer transcripts exacerbate conflicts [46,47]. This is likely because high RNAP density and increased gene length increase the likelihood of an encounter between elongating RNAPs and the replisome.

*Transcription termination*: In bacteria, there are two mechanisms of termination: intrinsic termination and factor-dependent termination (Reviewed in [48]). During intrinsic termination, a sequence-specific signal promotes destabilization and consequent dissociation of RNAP. Specifically, a uridine-rich sequence promotes RNAP pausing, allowing for subsequent formation of a GC rich terminator hairpin structure within the RNAP exit channel. This ultimately promotes weakening of the RNA-DNA hybrid contacts within the transcription bubble, facilitating release of RNAP from both the DNA and RNA. In factor-dependent termination, the hexameric helicase protein Rho binds to the nascent RNA transcript, translocates on RNA, ultimately associating with RNAP. Rho utilizes its motor force to promote release of RNAP from DNA and RNA, either by driving RNAP forward on DNA and collapsing the transcription bubble [49], or by shearing the RNA within the RNAP exit channel [50].

There is evidence that efficient termination is important in reducing replication-transcription conflicts, as inhibition of Rho promotes conflict induced genomic instability [29,51]. Studies in *Saccharomyces cerevisiae* reveal that the transcription termination factor Sen1 helicase helps mitigate transcription-induced genomic instability [52]. Currently, no studies have determined whether conflicts occur at intrinsic termination sites in bacteria. Moreover, the dynamics and conformational changes that RNAP undergoes during termination are still largely unclear [48]. A more precise understanding of what the state of a terminating RNAP is when it encounters the replisome would help clarify if conflicts between the replisome and terminating RNAPs are fundamentally different from those with elongating or initiating RNAPs.

## 4 R-loops and conflicts

Work in both prokaryotes and eukaryotes has highlighted the importance of R-loop formation in exacerbating the consequences of replication-transcription conflicts [[11], [12], [13],^53^,^54^]. R-loops are transcription dependent, three-stranded nucleic acid structures generally consisting of nascent mRNA re-annealed to its complementary coding strand along with the displaced single non-coding DNA strand [55]. These stable structures form preferentially at head-on conflict regions, causing severe replication stalling and genomic instability [13,53] (reviewed in [56]). It thus seems that R-loop formation helps at least partially explain why head-on conflicts are more severe than co-directional conflicts. Exactly why R-loops exist at greater levels at head-on conflicts remains unclear. Additional factors that may promote R-loop formation include GC richness (including G-quadruplexes [57,58] and G-rich pause sites [59]); and in eukaryotes, the state of chromatin [60]. Therefore, particular regions in genomes may be potential hotspots for conflicts. Interestingly, it has been suggested that R-loops can promote stalling or backtracking of upstream RNAPs [29]. This could potentially lead to an array of arrested RNAPs, which could further strengthen the impediment faced by the replisome.

Overall, these findings paint a complex, nuanced understanding of how RNAP can become a significant obstacle for the replisome. In particular, the state of RNAP and transcription are critically important for the severity and consequences of an encounter with replication. However, much of what we know regarding the state of RNAP and conflicts are from studies utilizing specific conditions such as UV damage as well as engineered reporter systems. Greater insight into how factors such as RNAP pausing, sequence context, and translation efficiency alters the severity and/or presence of conflicts at endogenous sites would further our understanding of conflicts.

## 5 Mfd and replication transcription conflicts: an enigma

*An overview of Mfd*: RNAP faces an array of obstacles during transcription and it is therefore not surprising that multiple proteins can help RNAP deal with such impediments. One of the well-studied factors that helps with stalled RNAP in bacteria is the highly conserved Mfd translocase protein. Mfd is classically described as a DNA repair protein that links nucleotide excision repair (NER) and transcription via the transcription-coupled repair (TCR) pathway (reviewed in [17,61,62]), leading to preferential repair of the transcribed strand [63,64]. In the context of TCR, Mfd recognizes stalled RNAP (canonically due to a bulky DNA lesion such as a cyclopyrimidine dimer), is loaded onto DNA and subsequently utilizes its ATPase and translocase activity to displace stalled RNAP from DNA [65,66]. This exposes the offending lesion to NER proteins, which Mfd recruits to the site of damage via binding to UvrA [67]. TCR is a highly conserved mechanism that exists throughout all domains of life. Mutations of the functional homolog of Mfd in humans, CSB, leads to the Cockayne Syndrome (CS), a debilitating neurodegenerative disease as well as UV-sensitive syndrome [68,69]. Interestingly, the severe developmental defects in CS are due to a role of CSB that appears to be unrelated to TCR [61].

More recent work has broadened the role of Mfd outside of its DNA repair function and has provided new mechanistic insights. Various *in vitro* and *in vivo* studies show that Mfd utilizes its translocase activity to move extensively backtracked RNAP forward as well as to remove arrested RNAPs at a wide variety of obstacles, ranging from protein roadblocks to sites of nucleotide starvation [[70], [71], [72], [73], [74], [75]]. Recent *in vitro* work shows that Mfd translocates autonomously (independent of RNAP) upon binding to DNA [71]. Consequently, Mfd is capable of recognizing RNAP arrested downstream of the initial site where it was loaded. Mfd’s function on arrested RNAP depends on the nature of the roadblock as it can either rescue severely backtracked RNAPs or promote transcription termination if the obstacle cannot be overcome [71]. Additionally, single-molecule microscopy imaging of Mfd reveals that it binds to DNA *in vivo* in the absence of exogenous stressors [76], corroborating *in vitro* findings and providing further evidence that Mfd may function as a fundamental transcription processivity factor outside of a RNAP-blocking lesion, such as a photoproduct from UV irradiation. This is consistent with evidence that CSB promotes transcription elongation [77,78] in mammalians, in addition to its role in TCR.

Given that the replication fork is a significant obstacle to RNAP processivity, it is logical to examine the role of Mfd in reducing the severity of replication-transcription conflicts. Intriguingly, the answer seems complex.

*A relationship between Mfd and conflicts?* Trautinger et al. [18] provided the initial genetic evidence for the role of Mfd in replication-transcription conflicts. The authors showed that a deletion of Mfd (in addition to other factors that deal with stalled RNAP) sensitized cells to UV damage when cells were additionally deficient in DNA repair [18]. This effect was eliminated in the presence of a mutant RNAP (rpo*35), which destabilizes RNAP elongation [18]. The authors propose a model whereby UV stalled RNAP complexes promote genomic instability upon encounters with replication. In this context, RNAP associated factors such as Mfd seem to be generally important for maintaining genomic stability.

**Fig. 1:**
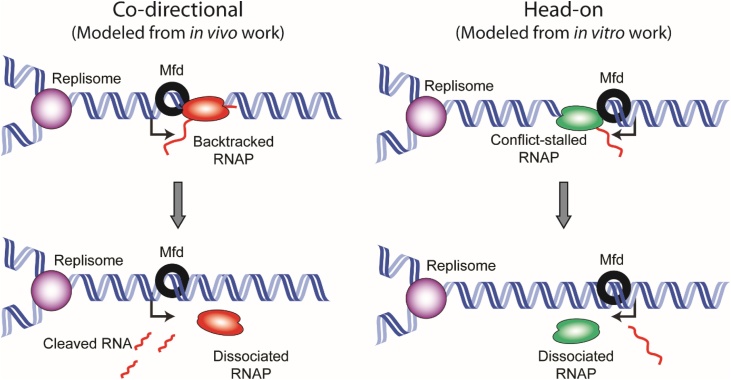
Models of conflict resolution by Mfd. The left panel shows Mfd in resolution of a co-directional conflict, cause by a backtracked RNAP. This model is based on the observations by Dutta et al. [29], *in vivo*, in *E. coli*. Mfd does not appear to be important for conflict resolution, at least in the context of backtracked RNAP, *in vivo*. The right panel shows a model for resolution of head-on conflicts, which is based on work by Pomerantz and O’Donnell [79]. The *in vitro* work placed a single RNAP ahead of the fork, and in that context, Mfd helped resolve the conflict, but only in the head-on orientation.

Pomerantz and O’Donnell undertook the first mechanistic study directly looking at the role of Mfd in conflicts. The authors of this study reconstituted the bacterial replisome *in vitro* and loaded it head-on to an arrested RNAP complex on a linear double stranded DNA fragment. They found that replication across the linear fragment was inhibited by the presence of the head-on RNAP. The addition of Mfd into the system significantly reduced the replication stall generated by RNAP [79]. The authors concluded from their findings that Mfd activity helps promote replication through head-on transcription units, likely by displacing RNAP from DNA (Fig. 1). Previous work from the same group suggested that Mfd is not necessary for resolution of co-directional conflicts due to the ability of replication to reinitiate using an mRNA primer [80].

Shortly after these findings, Dutta, et al. [29] developed an *in vivo* system to investigate genomic instability at replication-transcription conflict regions as well as the importance of various RNAP associated factors in mitigating DNA damage at conflict sites. Using a plasmid based system, the authors measured the presence of DSBs at head-on and co-directional conflict sites and found that they were dependent on RNAP backtracking [29]. While conflicts induced DSBs in both orientations, Mfd was able to reduce DSBs only when the conflict was co-directional (Fig. 1) [29]. These findings contrast with the findings of Pomerantz and O’Donnell, which implicated Mfd in helping replisome progression through head-on but not co-directional conflict regions.

**Fig. 2:**
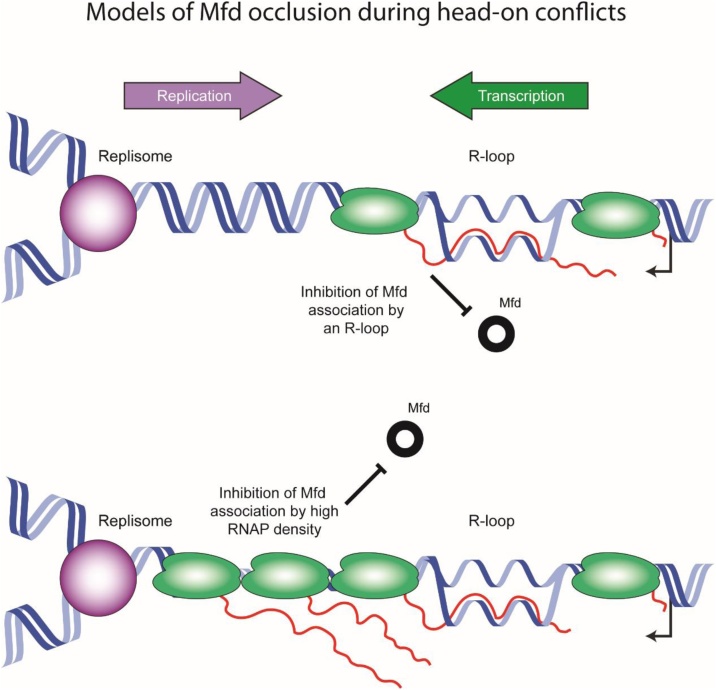
Models for why Mfd may not resolve head-on conflicts in the *in vivo* context. There are two fundamental differences between the *in vitro* work, where Mfd was observed to resolve head-on conflicts, and what takes place *in vivo* at regions of head-on transcription. Lang et al. [13] have shown that R-loops accumulate at head-on conflict regions. It is possible that the R-loops prevent Mfd binding to RNAP and occlude Mfd from the conflict region (Top). However, in contrast to the *in vitro* set up, the majority of genes *in vivo* are transcribed by more than a single RNAP. It is very likely that the 25bp gap needed for Mfd to sit on DNA is not available in the head-on conflict regions when RNAP density is high at a given gene (Bottom).

Why Mfd is unable to reduce DSBs at head-on conflicts remains unclear. Perhaps *in vivo,* Mfd is unable to access or remove RNAP at severe head-on conflicts. Mfd is known to require access to at least 25 basepairs of DNA upstream of RNAP to bind to the protein [74]. It is possible that at a highly expressed head-on gene, tightly arrayed RNAPs block access of Mfd (Fig. 2). Additionally, recent work from Lang, et al. [13] show that R-loops preferentially form at head-on conflict sites. Since R-loops likely form upstream of elongating RNAPs, they may also inhibit Mfd accessibility (Fig. 2). Lastly, it is critical to note that the effect of Mfd in relieving DSBs at co-directional conflicts is in the presence of a strong transcription arrest via an engineered protein roadblock. It is not clear whether Mfd relieves DSBs at other co-directional conflict sites (e.g. pause sites, endogenous roadblocks, rRNA genes).

## 6 Is Mfd a critical conflict resolution factor?

Many Replisome-RNAP conflict resolution factors are either essential proteins or become essential upon induction of a head-on conflict. Although Mfd can reduce DSBs at co-directional conflicts and help replisome progression *in vitro*, evidence suggests that, in contrast to all other Replisome-RNAP conflict resolution factors, Mfd does not help cells survive the consequences of conflicts. Boubakri, et al. [11] and De Septenville, et al. [9] show that inversion of rRNA genes to the head-on orientation in *E. coli* require the activity of the DinG, Rep, and UvrD helicases as well as the RecBC double-stranded end processing enzymes for cell viability. However, deletion of Mfd, either alone or in conjunction with the aforementioned helicases, does not affect cell viability [9]. Mfd is one of various RNAP accessary factors (e.g. Rho, GreA/GreB – see below) that may be capable of dealing with the consequences of stalled RNAPs at replication-transcription conflicts. Perhaps this redundancy in the system explains the lack of cell viability phenotypes associated with an Mfd deletion in the presence of severe replication-transcription conflicts. However, this model seems unlikely given that these various proteins have very different modes of action compared to Mfd.

## 7 Mfd as a mutagenic factor at conflict regions

One of the various consequences of replication-transcription conflicts is DNA mutagenesis [46,81,82]. Utilizing chromosome reporters in *Bacillus subtilis*, initial work by Paul et al. [46] revealed that genes oriented head-on to replication had a higher rate of spontaneous mutagenesis relative to the same genes oriented co-directionally. A follow up study by Million Weaver et al. [81] discovered that the enhanced mutation rate of head-on genes was actually *dependent* on Mfd, as deletion of Mfd reduced mutation rates of the head-on genes studied. Additionally, the authors found an epistatic relationship between Mfd and other factors of the TCR pathway, and proposed a model by which TCR activity at head-on conflicts promoted error-prone DNA repair [81]. This work suggests that Mfd acts at head-on conflicts but does so in a mutagenic manner. Given that Mfd promotes mutagenesis at these sites, it is worth considering a model where Mfd *impedes* or *slows* resolution of head-on conflicts to some degree. Mfd could increase mutagenesis by delaying an unknown step during conflict resolution. For example, previous work suggests that Mfd can impede the activity of helicases in the context of DNA repair [83], so it is conceivable that Mfd activity at head-on conflicts also impedes the activity of other helicases and/or repair proteins that promote conflict resolution, thus increasing mutagenesis.

Interestingly, additional work shows that Mfd promotes mutagenesis under various other conditions, including stationary-phase mutagenesis [84,85], cellular starvation conditions [86], as well as in the context of antimicrobial resistance (AMR) [[87], [88], [89]]. While none of these studies directly address the potential role of replication-transcription conflicts in Mfd-driven mutagenesis, Mfd requires interactions with RNAP and UvrA to promote AMR development [87]. This suggests that the canonical role of Mfd in TCR may be the mechanism by which Mfd promotes mutagenesis. In general, it seems that Mfd promotes transcription-associated mutagenesis at many different endogenous loci that may be prone to encountering conflicts.

Further insights into Mfd’s mechanism of mutagenesis will allow for a more comprehensive understanding of how Mfd’s specific activity at conflict sites (as well as other sites where RNAP is arrested) is linked to its ability to promote mutagenesis and evolution.

## 8 What happens to transcription in a conflict?

Replication-transcription conflicts have deleterious consequences on replication, but little is known about the consequences of conflicts on transcription. Lang, et al. [13] provided the first evidence that transcription may be compromised during head-on conflicts, but the mechanism of transcription attenuation at conflicts remains unclear. Additionally, the role of RNAP associated factors such as Mfd on transcription dynamics at conflict sites has not been studied. Evidence does suggest that Mfd is capable of regulating transcription given that *in vitro*, Mfd terminates transcription during RNAP arrest. Furthermore, *in vivo* evidence implicates Mfd in relieving transcriptional roadblocks and subsequently *decreasing* transcription at various sites in the *B. subtilis* genome. These findings suggest that Mfd helps regulate transcription when RNAP encounters a roadblock. Whether or not Mfd functions in a similar manner at conflict regions remains to be seen. Given that a critical function of Mfd is to deal with consequences of halted transcription elongation complexes, this is an interesting and critical avenue for further study.

## 9 Additional RNAP associated factors and conflicts

While we have predominantly focused on reviewing the activity of Mfd at replication-transcription conflict regions, it is important to discuss other proteins that help rescue arrested RNAPs, as these proteins may also have fundamental roles as Replisome-RNAP conflict resolution factors.

GreA and GreB are critical proteins that also rescue backtracked RNAPs [29]. Biochemically, GreA and GreB work identically and function by cleaving the nascent RNA that has extruded from the RNAP exit channel during backtracking, helping stimulate transcription elongation [90,91]. Genetic evidence implicates GreA/GreB in resolution of conflicts [92], including reduction of conflict-induced DSBs [29]. Much like Mfd, Dutta and colleagues found that GreA /GreB reduced conflict-induced DNA breaks only in the co-directional orientation [29]. Although both Mfd and GreA/GreB perform antibacktracking functions, the relative importance of these proteins in conflict resolution remains unclear.

In *E. coli*, the transcription factor DksA functions to alleviate conflicts between replication and RNAPs that are arrested due to amino acid starvation [92]. DksA functions both to modulate transcription initiation [93] as well as to promote transcription elongation [94]. Deletion of DksA under starvation conditions induced replication arrest, and this effect was rescued by reducing transcription with a drug and by utilizing a mutant version of RNAP that is less prone to conflicts [92]. In this context, DksA seems to function as a transcription elongation factor, although the precise mechanism by which the recovery of transcription elongation by DksA reduces conflict severity is unclear. Additionally, DksA is not as conserved as Mfd, and it is absent in both *B. subtilis* and other Gram-positive species, highlighting that different species may use divergent strategies to reduce conflict severity.

The transcription termination factor Rho also helps reduce conflict severity. Chemical inhibition of Rho with the antibiotic bicyclomycin increased the presence of DSBs at conflict regions [51], but according to Dutta and colleagues this effect is specific to co-directional conflicts [29]. Depletion of Rho also stimulates persistent R-loop formation genome wide [95]. Given that R-loops accumulate at head-on conflicts [13,53], it is possible that Rho reduces conflict severity by reducing R-loop formation. Excessive R-loop formation however is specific to head-on conflicts, therefore, it is unclear why and how R-loop removal proteins would be important in the aforementioned studies only in the co-directional orientation.

## 10 Concluding remarks

The field of replication-transcription conflicts has made significant advances in understanding both the consequences of conflicts as well as the role of many Replisome-RNAP conflict resolution factors in minimizing these damaging events. Such factors affect a diverse range of processes, ranging from R-loop resolution, to RNAP antibacktracking, to replication restart. But fundamentally, these proteins share one thing in common: they help mitigate and reduce the life-threatening consequences of conflicts. While we have made great progress in identifying the importance of these proteins, many critical and in particular mechanistic questions regarding how they function in conflicts remain unanswered.

One such question is how the enigmatic Mfd translocase helps deal with conflicts, if at all. While Mfd promotes replication progression at a head-on conflict *in vitro*, *in vivo*, in some studies, Mfd mitigates some consequences of only co-directional and not head-on conflicts. Additionally, Mfd promotes mutagenesis at head-on genes, suggesting that it does indeed play a role in such conflicts *in vivo,* but this involvement might actually be counter-productive and potentially even detrimental for conflict resolution. Given that Mfd increases mutagenesis at conflict regions, it is indeed possible that Mfd promotes genomic instability at head-on conflict regions. Whether this effect is a trade-off for other functions of Mfd during these encounters, such as efficient transcription elongation, or an inhibition of other Replisome-RNAP conflict resolution factors, remains to be seen. As we continue to gain greater insights into differences between head-on and co-directional conflicts, as well as insights into the mechanism of Mfd’s activity, a clearer understanding of how and why Mfd functions at conflict regions will arise.

Lastly, the undiscovered frontier in the field of conflicts is understanding how transcription is altered when it encounters the replisome. Studies on RNAP accessory factors, such as Mfd, in mitigating conflicts have exclusively looked at the role of these proteins in helping reduce replication stress. Given the biochemical activities of this class of proteins, it is quite likely that they alter transcription, but the consequences on gene expression and RNAP dynamics at conflict regions still requires further research. Such work will provide novel insight into the consequences of conflicts.
